# Immobilization of *Ochrobactrum* sp. on Biochar/Clay Composite Particle: Optimization of Preparation and Performance for Nitrogen Removal

**DOI:** 10.3389/fmicb.2022.838836

**Published:** 2022-03-02

**Authors:** Pengfei Sun, Xiao Huang, Yixiao Xing, Wenlong Dong, Jianghua Yu, Jie Bai, Weiyan Duan

**Affiliations:** ^1^Ministry of Natural Resources, Fourth Institute of Oceanography, Beihai, China; ^2^Key Laboratory of Tropical Marine Ecosystem and Bioresource, Ministry of Natural Resources, Beihai, China; ^3^Guangxi Beibu Gulf Key Laboratory of Marine Resources, Environment and Sustainable Development, Beihai, China; ^4^Jiangsu Key Laboratory of Atmospheric Environment Monitoring and Pollution Control, Collaborative Innovation Center of Atmospheric Environment and Equipment Technology, School of Environmental Science and Engineering, Nanjing University of Information Science and Technology, Nanjing, China; ^5^Shandong Marine Forecast and Hazard Mitigation Service, Qingdao, China; ^6^College of Environmental Science and Engineering, Ocean University of China, Qingdao, China; ^7^Ocean College of Hebei Agricultural University, Qinhuangdao, China

**Keywords:** biochar/clay composite particle, calcined temperature, ammonia-oxidizing bacteria, immobilization, *Ochrobactrum* sp.

## Abstract

The objective of this study was to prepare biochar/clay composite particle (BCCP) as carrier to immobilize *Ochrobactrum* sp. to degrade ammonia nitrogen (NH_4_^+^-N), and the effects of calcined program and immobilizing material were investigated. Results reflected that the parameters were as follows: calcined temperature 400°C, heating rate 20°C min^–1^, and holding time 2 h, and the adsorption capacity could reach 0.492 mg g^–1^. Sodium alginate/polyvinyl alcohol, as embedding material, jointed with NH_4_^+^-N adsorption process and then degraded by *Ochrobactrum* sp. with 79.39% degradation efficiency at 168 h. Immobilizing *Ochrobactrum* sp. could protect strain from high salt concentration to achieve the exceeding degradation efficiency than free bacteria, but could not block the impact of low temperature.

## Introduction

Liaohe Estuary Wetland (LEW) owned the functions of regulating climate, alleviating flood peak, providing habitat for wildlife, and protecting biodiversity, and crab farming is the main industry there (Lin et al., 2016). Hence, serious ammonia nitrogen (NH_4_^+^-N) pollution was caused by excessive crab feed and contributed to the eutrophication in LEW (Hina et al., 2015).

A kind of biochar/clay composite particle (BCCP) absorbing NH_4_^+^-N was prepared with waste biochar and clay in LEW by previous studies and demonstrated that its removal effectively related to the ratio of materials, and the dosage of crosslinking agent and pore-forming agent (Huang et al., 2020). In fact, the calcined temperature and program of BCCP are also the key parameters restricting and affecting its adsorption performance and adsorption capacity depending on the changing of adsorption site quantity and adsorption material structures (Mandal and Mayadevi, 2008; Feng et al., 2013; Sun et al., 2015). Lin et al. (2009) found that the phenol adsorption capacity by nano-hydroxyapatite powder from aqueous solution reduced obviously when it was calcined at high temperature. For TiO_2_, the organic moieties were destroyed by high calcination temperature and affected the adsorption performance (Feng et al., 2020). On the contrary, Yan et al. (2018) prepared porous diatomite microspheres with different calcined temperatures, and concluded that the production was amorphous at 800°C and crystallized into crystobalite at 1,000°C. Nevertheless, whether a relationship between calcined temperature and NH_4_^+^-N adsorption capacity of BCCP exists or not needs to be further researched.

NH_4_^+^-N adsorption process is only the transfer of NH_4_^+^-N without complete conversion by ammonia-oxidizing bacteria (AOB). For LEW, the harsh environmental conditions of low temperature in winter and high salinity reduced the biological removal efficiency for NH_4_^+^-N. Therefore, screening high-efficiency degradation bacteria is a necessary method, and a previous study confirmed that an effective conversion process for NH_4_^+^-N could be achieved by salt- and cold-tolerant AOB under high-salt and low-temperature condition (Huang et al., 2017). Nevertheless, the application of high-effectivity degrading strains in a large-scale watershed faces an inevitable problem, i.e., the dilution of tide for using highly efficient AOB, which results in more difficult and inefficient application of traditional adsorption materials and biotechnology.

The immobilization technology of high-efficiency degradation bacteria is to fix the bacteria on a carrier, so as to solve the problem that the free high-efficiency degradation bacteria are washed away in the dynamic river. Hence, this technology is a potential application for wetland environmental restoration. Some previous studies have shown that immobilized strains could effectively remove reactive dyes, mineralize Ca^2+^ and Mg^2+^, and remove manganese (Reddy and Osborne, 2020; Yan et al., 2020; Atcharaporn et al., 2020). Meanwhile, whether this technology can maintain the degradation performance for salt- and cold-tolerant AOB converting NH_4_^+^-N and resist low temperature and high salt environment is worth discussing.

Therefore, the purpose of this study is to propose a method that can be applied to remove NH_4_^+^-N in LEW. Based on the previous research, BCCP was prepared and the influence of calcined temperature and program on its adsorption performance was discussed. Then, immobilized AOB was explored to investigate the contribution of different immobilization methods on NH_4_^+^-N degradation. Finally, salt- and cold-resistance characteristics of immobilization were further studied to deepen the application value of immobilization technology.

## Materials and Methods

### Biochar, Clay, and Ammonia-Oxidizing Bacteria

The reed straw selected was washed with deionized water and dried at 105°C for 24 h in an open crucible to remove the surface magazine. Then it was crushed with a micro plant crusher (Beijing Weiye, Z102), screened to obtain 0.85 mm reed powder, and placed in a quartz tube inside a tube furnace to produce the biochar through slow pyrolysis in a N_2_ environment at 600°C for 3 h, respectively. The biochar samples were washed with deionized water several times to remove impurities, and then grinded into 0.15 mm powder and sealed in a container for further testing. The detailed information of the biochar characteristics is shown in Huang et al. (2020), and the composition of C, H, O, and N were 72.5, 4.18, 18.32, and 0.67%, respectively. The proportion of ash was 12.31%.

The clay was placed in an open crucible at 105°C for 24 h. Then it was crushed with a micro plant crusher (Beijing Weiye, Z102) and screened to obtain 0.15 mm clay powder.

The AOB was isolated from LEW with the characteristics of cold and salt tolerance and similar to the branch *Ochrobactrum* sp. The obtained 16S rDNA sequence of HXN-1 strain was registered in GenBank under accession numbers KP276672, and the characteristics of *Ochrobactrum* sp. and phylogenetic tree are listed in Huang et al. (2017). The details were as follows: catalase test (−), starch hydrolyzing enzyme test (+), citrate utilization test (+), MR test (−), glucose fermentation test (−), VP test (−), and indole test (−). The NH_4_^+^-N removal rate by *Ochrobactrum* sp. exceeds 60% at 15°C and 20‰ condition.

### Preparation of Biochar/Clay Composite Particle

**FIGURE 1:**
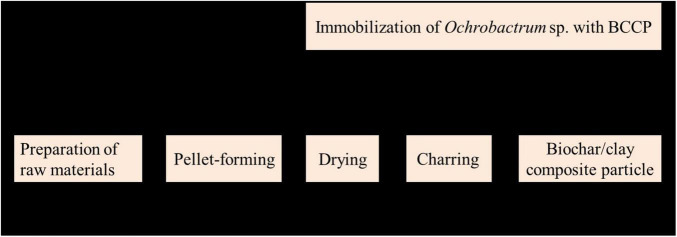
Calcined program of BCCP and immobilization of *Ochrobactrum* sp.

The optimum preparation formulation of BCCP and the proportion of raw material obtained in a previous study are demonstrated as follows: 15% biochar, 79% clay, 3% Na_2_SiO_3_, and 3% NaHCO_3_ (v/v) (Huang et al., 2020). These materials were mixed, placed in a disc-type ball-making machine (BY-300; TianZhuo, Zhengzhou) to produce BCCP with a particle size of 8∼10 mm, and dried at 45°C for 6 h in a constant temperature drying oven. The calcined process was slow pyrolysis in a N_2_ environment at 400, 450, 500, 550, 600, and 700°C for 3 h, respectively. Besides, the heating rate and holding time were optimized and their optimization scope was 5∼20 °C min^–1^ and 1∼4 h, respectively. The firing process is shown in Figure 1. The calcined process orthogonal test level of BCCP is demonstrated in Table 1.

**TABLE 1:** Calcined process orthogonal test level of BCCP.

Levels	Calcined temperature (°C)	Heating rate (°C min^–1^)	Holding time (h)	Empty column
1	400	5	1	1
2	400	10	2	2
3	400	15	3	3
4	400	20	4	4
5	500	5	2	3
7	500	10	1	4
7	500	15	4	1
8	600	20	3	2
9	600	5	3	4
10	600	10	4	3
11	600	15	1	2
12	600	20	2	1
13	700	5	4	2
14	700	10	3	1
15	700	15	2	4
16	700	20	1	3

### Adsorption Experiment of Biochar/Clay Composite Particles for Ammonia Nitrogen

#### Adsorption Batch Experiment

BCCP (1.0 g) calcined with six different temperatures were put into a 50-ml flask with pure NH_4_^+^-N solution and shaken at 150 r min^–1^ for 300 min at 25°C. Samples were collected at 5, 10, 20, 40, 60, 90, 120, 150, 180, 240, and 300 min. The samples were filtrated by 0.45-μm RC membrane to determine NH_4_^+^-N concentration.

The adsorption capacity during the adsorption period was calculated by Equation (1):


(1) $$q_{t}=\left(C_{0}-C_{t}\right)\,\frac{\text{V}}{\text{m}}$$

where *q*_*t*_ is the amount of NH_4_^+^-N during the adsorption time (mg kg^–1^); *C*_0_ and *C*_*t*_ (mg L^–1^) are the initial NH_4_^+^-N concentrations and different time residual concentration, respectively. V is the volume of reaction system (L), and m is the mass of adsorbent (g).

#### Adsorption Kinetics

The data coming from adsorption batch experiment were fitted with pseudo-first- and pseudo-second-order models and intraparticle model, the expressions as following Equations (2)–(4):


(2) $${\text{q}}_{\text{t}}={\text{q}}_{\text{e}}\,\left(1-{\text{e}}^{-K_{1}\,\text{t}}\right)\;\left(\mathrm{Firstorder}\right)$$

(3) tqt=1K2⁢qe2+tqe  (Secondorder)

(4) $${\text{q}}_{\text{t}}={\text{K}}_{\text{p}}\,\sqrt{\text{t}}+\text{C}\;\left(\mathrm{Intraparticlemodel}\right)$$

where q_e_ (mg⋅g^–1^) is the adsorbed amounts of NH_4_^+^-N by the BCCP at equilibrium time, and q_t_ is the adsorbed amount at a given time interval (t). K_1_ and K_2_ are the rate constants for the pseudo-first- and pseudo-second-order models, respectively. Kp is the intraparticle diffusion rate constant (mg⋅g^–1^ min^1/2^), and C (mg g^–1^) is a constant that reflects the boundary layer effect. A plot of q_t_ against t^1/2^ gave a linear relationship from which the Kp value was determined from the slope and C as the intercept.

#### Adsorption Isotherms

Freundlich and Langmuir equations were used to fit the adsorption isotherms of BCCP with different calcined temperatures, and the equation is given by


(5) $$\text{log}\,q_{e}=\text{log}\,K_{F}+\frac{1}{n}\,l\,o\,g\,C_{e}\,\left(\mathrm{Freundlichequation}\right)$$

(6) $$q_{\text{e}}=\frac{q_{\text{max}}\,K_{\text{L}}\,C_{\text{e}}}{1+K_{\text{L}}\,C_{\text{e}}}\;\left(\mathrm{Langmuirequation}\right)$$

where *q*_*e*_ (mg⋅g^–1^) is the amount of NH_4_^+^-N adsorbed by the BCCP at equilibrium time, and *q*_*max*_ (mg g^–1^) and *K*_*L*_ (L mg^–1^) are Langmuir constants that indicate the maximum adsorption and relative binding energy of BCCP, respectively. *K*_*F*_ and *n* are Freundlich constants that measure the relative NH_4_^+^-N adsorption capacity and adsorption intensity of BCCP, respectively, while *C*_*e*_ (mg L^–1^) denotes the equilibrium concentration of NH_4_^+^-N remaining in solution after adsorption is complete.

### Immobilization of *Ochrobactrum* sp. With Biochar/Clay Composite Particle

The AOB strain HXN-1 (*Ochrobactrum* sp.) used in this study was enriched with culture medium to OD_600_ = 0.6. The prepared BCCPs were soaked into high-efficiency degrading bacteria (OD_600_) for 24 h and afterward were transferred into the embedding solution for immobilization.

Two immobilization groups were set with sodium alginate (SA) and polyvinyl alcohol (PVA). (1) SA immobilization group: 2% SA aqueous solution and 2% CaCl_2_ aqueous solution were mixed and autoclaved at 121°C for 30 min. (2) SA/PVA immobilization group: 2% SA and 12% PVA aqueous solutions were prepared according to the aforementioned method. For the BCCP adhesive, two kinds of embedding liquid were transferred into 2% CaCl_2_ solution and saturated boric acid–2% CaCl_2_ solution, respectively, and afterward placed in a 4°C refrigerator for 24 h.

### Batch Experiment of Ammonia Nitrogen Degradation by *Ochrobactrum* sp.

#### Influence of Immobilization Material and Bacteria on Ammonia Nitrogen Degradation

HXN-1was made into gel particles by the method of 2.4 and named SA-B and SA/PVA-B and the blank gel particles were named SA-C and SA/PVA-C. SA and PVA as base material to immobilize *Ochrobactrum* sp. was named SA/PVA-B, and as control group without adding *Ochrobactrum* sp. was named SA/PVA-C. The aforementioned gel particles were put into 100 ml of 50 mg L^–1^ NH_4_^+^-N medium, placed in a shaking incubator at 25°C, 180 r min^–1^ for 7 days, and NH_4_^+^-N concentration was measured daily. The medium characteristics were demonstrated in Huang et al. (2017). Free bacteria (FB) were used as a control group.

#### Influence of Salinity and Temperature on Ammonia Nitrogen Degradation

Six kinds of gel particles were, respectively, put into 100 ml of 50 mg L^–1^ NH_4_^+^-N solution with different salinities (0, 5, 15, 25, and 35‰) under 25°C condition. Also, the same six kinds of gel particles were with different temperatures (15, 20, 25, 30, and 35°C) with 5‰ salinity. All of these were placed in a shaking incubator and shocked with 180 r min^–1^ for 10 days and NH_4_^+^-N concentration was measured daily. Free bacteria were used as a control group.

### Analytical Method

Fourier-transform infrared spectroscopy (FTIR) spectra were recorded between 400 and 4000 cm^–1^ on a Nicolet 6,700 Fourier transform spectrometer. Clay, biochar, and BCCP were pelletized from a mixture of 1.5 mg dried sample with 200 mg KBr.

The water sample was filtered with a 0.45-μm filter membrane (Minisart RC 15), and the NH_4_^+^-N concentration was measured with a Nessler reagent. Each sample was measured in triplicate, and their average value was analyzed.

### Statistical Analysis

All experiment groups were set in three replicates, and the average values of each sample were calculated and showed in charts. Origin 8.6 software was used for drawing figures.

## Results and Discussion

### Effect of Calcined Program on Ammonia Nitrogen Adsorption by Biochar/Clay Composite Particle

To a certain extent, the control of calcined program (calcined temperature, heating rate, holding time) changes the surface structure of BCCP and then affects the adsorption performance for NH_4_^+^-N. Previous studies have shown that the adsorption performance of biochar was affected by calcined temperature and heating rate (Yakkala et al., 2013; Mahdi et al., 2018). Therefore, orthogonal experiment was used in this study to discuss the effect of calcined program on NH_4_^+^-N adsorption by BCCP, and the results are demonstrated in Table 2. Different BCCPs prepared by calcined temperature, holding time, and heating rate resulted in unequal NH_4_^+^-N adsorption capacity. The minimum adsorption capacity was 0.394 mg g^–1^, the maximum value was 0.454 mg g^–1^, and the average adsorption capacity was 0.424 mg g^–1^. The adsorption capacity of BCCP fluctuated with the increase of calcined temperature, and the best adsorption capacity appeared at 400°C; the adsorption capacity was 0.446 mg g^–1^, and the lowest was 500°C with the adsorption capacity reducing to 0.402 mg g^–1^.

**TABLE 2:** NH_4_^+^-N adsorption by BCCP with different calcined programs.

Levels	Calcined temperature (°C)	Heating rate (°C min^–1^)	Holding time (h)	Empty column	Result (mg g^–1^)
1	400	5	1	1	0.454
2	400	10	2	2	0.451
3	400	15	3	3	0.444
4	400	20	4	4	0.435
5	500	5	2	3	0.412
7	500	10	1	4	0.397
7	500	15	4	1	0.401
8	500	20	3	2	0.396
9	600	5	3	4	0.421
10	600	10	4	3	0.415
11	600	15	1	2	0.409
12	600	20	2	1	0.492
13	700	5	4	2	0.415
14	700	10	3	1	0.418
15	700	15	2	4	0.394
16	700	20	1	3	0.426
Average value 1	0.446	0.426	0.421	0.441	0.424
Average value 2	0.402	0.420	0.437	0.418	
Average value 3	0.434	0.421	0.420	0.424	
Average value 4	0.413	0.437	0.417	0.412	
Range analysis	0.044	0.017	0.020	0.029	
Primary relation	ACB				
Optimal scheme	A1B4C2 (calcined temperature: 400°C, heating rate: 20°C min^–1^, holding time: 2 h)				

Heating rate has a significant impact on the adsorption properties of BCCP and the adsorption capacity of BCCP decreased first and then increased with the increase of heating rate. The maximum adsorption capacity appeared at 20°C min^–1^. It can be interpreted that the increase of flexural strength decreased the loss tangent tan α when the temperature rose slowly. Meanwhile, the material was uniform with low porosity. However, too fast temperature rising would make it difficult to discharge the gas in BCCP and inhibited the reduction of porosity. Therefore, the adsorption capacity increased when the heating rate was from 10 to 20°C min^–1^.

The increase of holding time also promoted the adsorption capacity of BCCP first and then decreased. The maximum value appeared at 2 h with 0.437 mg g^–1^ adsorption capacity. The influence of holding time on adsorption properties of BCCPs mainly included two aspects, i.e., one was to stabilize the physical and chemical changes of materials, and the other was to homogenize the tissue structure. Too long holding time was not conducive to the formation of a strong skeleton, reduced mechanical properties, and caused glaze crack (Yang and Chow, 2019). Therefore, the holding time needed to be moderate, and the best holding time was 2 h in this study.

The primary relation of calcined temperature, heating rate, and holding time on NH_4_^+^-N adsorption was judged according to the magnitude of extreme difference; the primary and secondary sequences of the three ingredients were ACB, which proved that calcined temperature occupied the most prominent position, and then holding time and heating rate owned the least impact. Besides, it was concluded that A1B4C2 was the best preparation condition for BCCP, and the parameters were as follows: calcined temperature 400°C, heating rate 20°C/min, and holding time 2 h.

### Effect of Calcined Temperature on Ammonia Nitrogen Adsorption by Biochar/Clay Composite Particle

#### Ammonia Nitrogen Removal Performance

**FIGURE 2:**
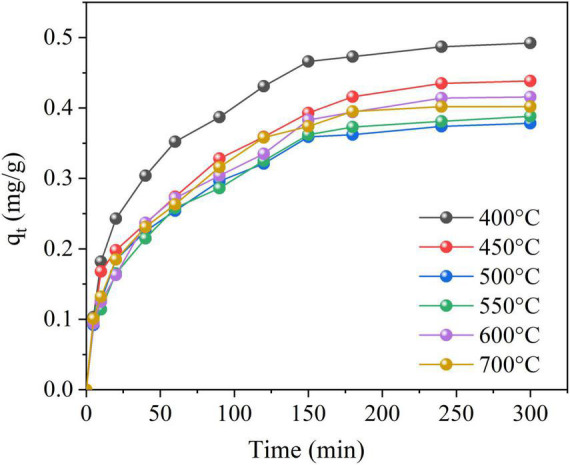
Adsorption capacity at different calcined temperatures.

NH_4_^+^-N adsorption performances from aqueous solution by six BCCPs with different temperatures were conducted, and the results are shown in Figure 2. The adsorption equilibrium time among six experimental groups was 180 min, and the NH_4_^+^-N removal efficiency by BCCP adsorption was about 29.4∼34.5%. The removal efficiency at 400°C group was better than that of the other four groups with the adsorption capacity of 0.473 mg g^–1^. However, the value decreased first and then increased with the increase of calcined temperature. The same phenomenon existed in the research of Chen et al. (2018) who found that the surface area and pore volume of bentonite increased to 56.09 and 0.0611 cm^3^ g^–1^ when the calcined temperature was 400°C, respectively, but sharply declined to 30.53 and 0.051 cm^3^ g^–1^ at 800°C. Yan et al. (2018) prepared a kind of porous diatomite microsphere by spray drying method and the methylene blue adsorption capacity and removal efficiency demonstrated the maximum values when the calcined temperature was 600°C, and decreased when the temperature rose. Ojeda-López et al. (2021) found that adsorbent/adsorbate interactions for CO_2_, CH_4_, and N_2_ were inversely proportional to calcined temperature (CMF-600 > CMF-700 > CMF-800) by the mean of the isosteric enthalpy of adsorption measurements.

This was because the pore volume and Brunauer–Emmett–Teller (BET) surface area reached maximum at some temperature and decreased further with the increase of calcined temperature (Kar and Equeenuddin, 2019). The organic compounds presenting in biochar or clay would condense on the surface of the particles, and clog the pores to decrease specific surface area after cooling with the increase of calcined temperature (Atkinson et al., 2010). Also, four forms of water existed in clay minerals (i.e., surface adsorbed water, pore adsorbed water, crystalline water combined with octahedral cations at the edge of pore, and cationic structural water combined with octahedral layer), and high temperature led to the adsorbed water, pore water, and bound water in the material lost when the temperature was less than 600°C, and the carbon in biochar and clay was oxidized. Meanwhile, the decomposition of NaHCO_3_ increased the pores and adsorption in the green body. The decrease of adsorption capacity from 500°C might be due to the fission of C400 biochar fired at 400°C with the temperature rising to 500°C, and the forming of ash adsorbed in the pores of BCCP and reduced its adsorption performance for NH_4_^+^-N. When the temperature exceeded 600°C, the water in BCCP evaporated and decomposed violently, and the pore structure was deformed, the porosity decreased, and the adsorption capacity decreased.

Many materials were reported to adsorb NH_4_^+^-N, such as slag, biochar, and coal slag balls. The NH_4_^+^-N adsorption behavior of slag was found in either neutral or alkaline conditions with 3.1 mg g^–1^ sorption capacity (Zhang et al., 2013). Vu et al. (2017) prepared biochar using corncob and the highest adsorption capacity was 22.6 mg g^–1^. However, Kong et al. (2021) reported that the biochar prepared from distilled spirit achieved lees adsorption capacity (5.92 mg g^–1^). Wang et al. (2016) prepared coal slag balls using modified coal slag and organic binder (PVA) and the NH_4_^+^-N adsorption capacity was only 0.09 mg g^–1^. The higher adsorption capacity of biochar depends on the large specific surface area and abundant adsorption sites (Li et al., 2018, 2019), and composition was also a key factor affecting the adsorption capacity. In this study, the high proportion of inorganic clay in BCCP resulted in small adsorption capacity.

#### Adsorption Kinetics and Isotherm

**FIGURE 3:**
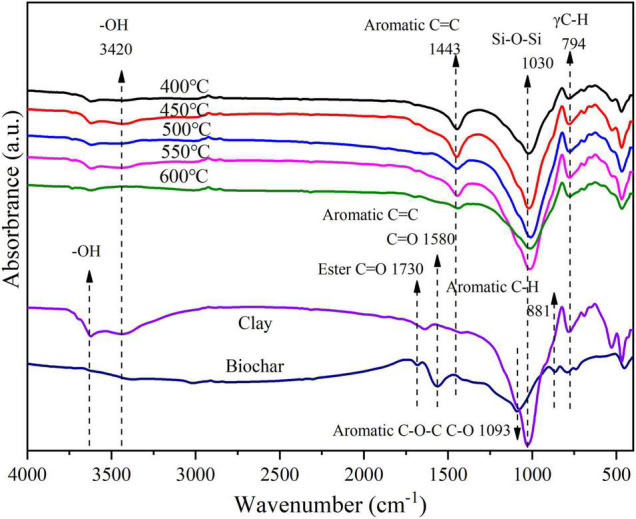
FTIR of raw materials and biochar/clay at different calcined temperatures.

Adsorption kinetics could be fitted by first-order kinetic model, second-order kinetic model, and intraparticle model, and all of them could well fit the NH_4_^+^-N adsorption process by BCCPs with different calcined temperature (Table 3). Comparing with first-order kinetic and intraparticle diffusion models, the second-order kinetic model was more suitable for describing the NH_4_^+^-N adsorption process by BCCPs, which was reflected by chemical adsorption processes including ion exchange among chemical bonds and adsorption process. Si–O–Si, –OH functional group on BCCP was involved in the reaction between chemical bonds during NH_4_^+^-N adsorption process (Figure 3). Yan et al. (2018) used porous diatomite microsphere to adsorb methylene blue and also found that the adsorption process followed the pseudo-second-order kinetic model.

**TABLE 3:** The adsorption kinetic parameters of BCCP under different calcined temperatures.

		Pseudo-first-order			Pseudo-second-order			Intraparticle diffusion	
T	q_e_	K_1_	q_eq_	*R* ^2^	K_2_	q_eq_	*R* ^2^	k_p_	*R* ^2^
(°C)	(mg g^–1^)	(min^–1^)	(mg g^–1^)		[g (mg min)^–1^]	(mg g^–1^)		[g (mg min^0.5^)^–1^]	
400	0.492	0.031	0.462	0.925	0.077	0.525	0.980	0.025	0.900
450	0.438	0.024	0.409	0.871	0.070	0.468	0.940	0.023	0.949
500	0.378	0.028	0.356	0.908	0.092	0.405	0.968	0.019	0.923
550	0.388	0.023	0.369	0.923	0.069	0.428	0.967	0.021	0.939
600	0.416	0.022	0.395	0.926	0.061	0.460	0.970	0.023	0.946
700	0.402	0.025	0.386	0.914	0.075	0.442	0.964	0.021	0.926

The adsorption equilibrium isotherm could reflect the distribution of adsorbate molecules in liquid and solid phases under an equilibrium state (Huang et al., 2020). Both Langmuir isotherm and Freundlich isotherm models could better fit NH_4_^+^-N adsorption by BCCP prepared with different temperatures (Table 4). However, Freundlich model with *R*^2^ = 0.941∼0.988 was more suitable than Langmuir model (*R*^2^ = 0.880∼0.946), and the conclusion was coincident with Yan et al. (2018). Adsorption site energy distribution characteristic and curvature in the isotherm could be responded by *n* in Freundlich model (Huang et al., 2020). The value of *n* was between 1 and 10 in this study, which proved that all adsorption processes of BCCPs with different calcined temperatures were preferential adsorption.

**TABLE 4:** The Langmuir and Freundlich adsorption isotherm constant of BCCP under different calcined temperatures.

T (°C)	Langmuir isotherm constants			Freundlich isotherm constants		
	q_m_	K_L_	*R* ^2^	1/n	KF	*R* ^2^
	(mg g^–1^)	(L mg^–1^)			(mg g^–1^) (L mg^–1^)^n^	
400	0.946	0.029	0.895	0.303	0.155	0.988
450	0.907	0.031	0.910	0.304	0.149	0.984
500	0.900	0.027	0.934	0.319	0.132	0.980
550	0.737	0.026	0.880	0.320	0.107	0.975
600	0.891	0.012	0.946	0.394	0.072	0.956
700	0.854	0.015	0.945	0.376	0.080	0.941

#### Fourier-Transform Infrared Spectroscopy Spectra of Biochar/Clay Composite Particles Under Different Temperatures

The FTIR is an essential technique to qualitatively determine characteristic functional groups of the adsorbents (Figure 3). The peak of IR curved at 1,030 cm^–1^ exhibited the introducing of Si–O–Si bonds on the BCCPs (Liu et al., 2012). The bands appearing below 1,100 cm^–1^ might be attributed to Si–O stretching, Si–O–Si bending, Si–O–Al bending, and Si–O–Mg bending vibrations (Chen et al., 2017). For the otherwise typical bands, the intensity of the OH stretch at approximately 3,420 cm^–1^ in the spectrum of clay was considerably larger than in the FTIR spectrum of the BCCP. The intensity of the CO_3_^2–^ stretch at approximately 1,440 cm^–1^ in the spectrum of BCCPs appeared depending on the addition of NaHCO_3_.

### Ammonia Nitrogen Degradation Performance by Immobilizing *Ochrobactrum* sp. on Biochar/Clay Composite Particle

#### Comparison of Immobilization Methods for Ammonia Nitrogen Degradation

**FIGURE 4:**
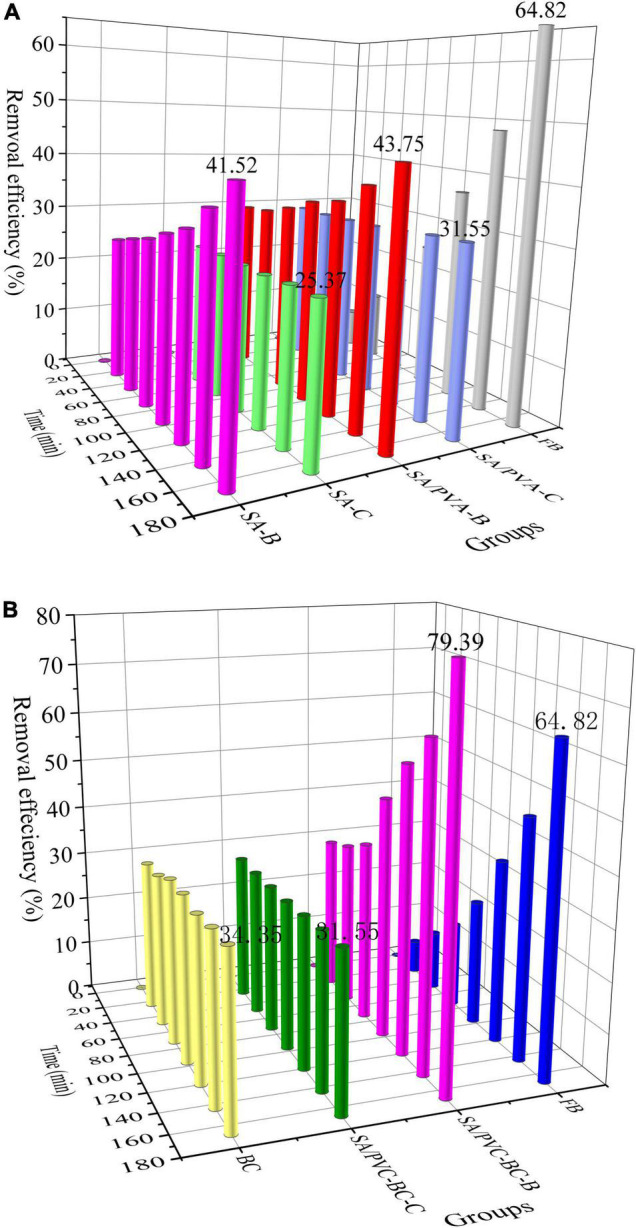
NH_4_^+^-N removal efficiency by immobilized *Ochrobactrum* sp. **(A)** Comparison of immobilization methods and **(B)** NH_4_^+^-N removal efficiency by immobilization of *Ochrobactrum* sp. on BCCP. FB, free *Ochrobactrum* sp. without any immobilization methods; SA-B, SA as base material to immobilize *Ochrobactrum* sp.; SA-C, SA as control group without adding *Ochrobactrum* sp.; SA/PVA-B, SA and PVA as base material to immobilize *Ochrobactrum* sp.; SA/PVA-C, SA and PVA as control group without adding *Ochrobactrum* sp.; BC, BCCP alone for adsorption as control group.

BCCP, as a carrier for microbial immobilization, is an effective method to ensure that the efficient flora continue its degradation efficiency in natural water and avoid the risk of free bacteria being dispersed to reduce its pollutant degradation performance (Huang et al., 2020). For maintaining its degradation efficiency, immobilization method becomes the main control factor restricting pollutant transformation in microbial immobilization process. In this study, immobilization method was studied first and the results are shown in Figure 4A. The immobilization of *Ochrobactrum* sp. exhibited preferable nitrogen removal capacities when ammonium chloride was used as the sole nitrogen source. During the initial stage of the experiment (the first 24 h), the gel particles (SA-C and SA/PVA-C groups) adsorbed NH_4_^+^-N from solution with high efficiency and their adsorption efficiencies were 22.59 and 29.59%, respectively, which were 10∼19% higher than that of the conclusion of Yan et al. (2020). Compared with two immobilization methods, on the contrary, the biodegradation performance of free *Ochrobactrum* sp. (FB group) at initial stage was very low (only 6.87% NH_4_^+^-N was transformed in 24 h) and NH_4_^+^-N removal efficiency reached 64.82% after 168 h. However, the value was 41.52 and 43.75% in SA-B and SA/PVA-B group, respectively, and they did not beat the FB group. Although the removal efficiency decreased, it could also be concluded that SA/PVA as immobilized material was more appropriate.

The fact that more nitrogen source and oxygen were obtained by free bacteria than immobilized bacteria prolonged the removal time by gel particle–immobilized bacteria (Yan et al., 2020). However, gel particles could provide stable micropores and protect cells from environmental changes and toxic substances (Hsieh et al., 2008; Hou et al., 2013). Zhang et al. (2021) prepared magnetic PVA–SA–diatomite composite carriers for immobilized microorganism and the highest NH_4_^+^-N removal rate reached 72.5% at 12 h. It was found that the adsorptions of NH_4_^+^-N by non-magnetic ingredients and Fe_3_O_4_ contributed 21.2 and 25.5%, respectively, and microorganism metabolism contributed 53.2%. Immobilized degrading bacteria in PVA–SA hydrogel bead was also reported to remove polycyclic aromatic hydrocarbons (PAHs) and the removal efficiency was around 77% in 96 h (Chen et al., 2021). Liu et al. (2019) investigated the effect of PVA–SA–cell cryogel bead–immobilized *Bacillus* sp. on the degradation of phenanthrene. The results indicated that the use of gel beads increased the number of adsorption sites to accelerate phenanthrene degradation.

#### The Improvement of Ammonia Nitrogen Degradation With Biochar/Clay Composite Particle as Carrier

On the basis of optimizing the immobilization method, BCCP was proposed as a carrier for *Ochrobactrum* sp. immobilization and the NH_4_^+^-N removal efficiency is demonstrated in Figure 4B. The strong adsorption of BCCP and gel made NH_4_^+^-N removal rate reach a high value on the first day. The efficiency of BC group (BCCP alone for adsorption as control group, 30.95%) was higher than that of SA/PVA-C group (29.94%), which reflected that the adsorption performance of gel particle was lower than BCCP and gel hindered the adsorption process of BCCP. Until 168 h, the removal efficiency was 34.35 and 31.55%, respectively, and little change was discovered during the process. On the contrary, the degradation efficiency of free *Ochrobactrum* sp. was dilatory and only 6.87% was achieved at the first 24 h, and increased to 84.82% at 168 h. This phenomenon reflected that biodegradation played its advantages. For SA/PVA-BC-B group, the degradation efficiency of NH_4_^+^-N kept higher than free *Ochrobactrum* sp. during the reaction process and it was up to 79.39% at 168 h, which exceeded 14.57% than free *Ochrobactrum* sp. group.

Compared with SA/PVA for microbial immobilization without adding BCCP, the results showed that BCCP as carrier to immobilize *Ochrobactrum* sp. could improve its degradation efficiency for NH_4_^+^-N. Because the porous structure of BCCP provided a larger surface area and a greater number of holes, it could store more substrate and promote microbial growth. The porous structure of BCCPs provides larger specific surface area and more pores, and can store more matrix and promote microbial growth (Chen et al., 2016).

#### Ammonia Nitrogen Degradation Mechanism of Immobilizing *Ochrobactrum* sp. With Biochar/Clay Composite Particle

**FIGURE 5:**
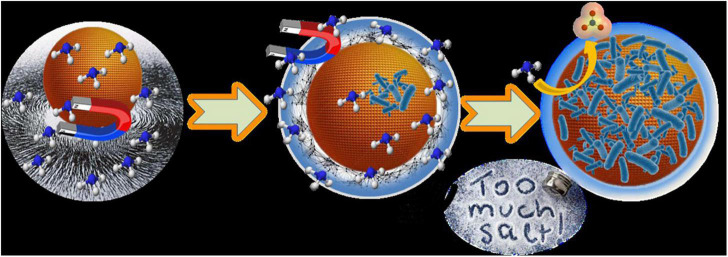
NH_4_^+^-N removal mechanism by immobilization of *Ochrobactrum* sp. on BCCP.

Based on the aforementioned research results, the NH_4_^+^-N degradation mechanism of immobilizing *Ochrobactrum* sp. with BCCP is demonstrated in Figure 5. The potential mechanisms were summarized as follows: the physical adsorption of gel and BCCP promoted NH_4_^+^-N accumulation rapidly on the surface of BCCP, and provided more appropriate condition for microbial degradation. However, the gel covering on BCCP had a certain resistance for BCCP adsorption. Besides, the *Ochrobactrum* sp. embedded in gel could degrade the high concentration of NH_4_^+^-N adsorbed on BCCP surface. The porosity of BCCP provides a necessary place for *Ochrobactrum* sp. growth and reproduction, and the adsorption driving force from BCCP promoted the biotransformation of *Ochrobactrum* sp.

### Effect of Salinity and Temperature on Nitrogen Removal

#### Effect of Salinity

**FIGURE 6:**
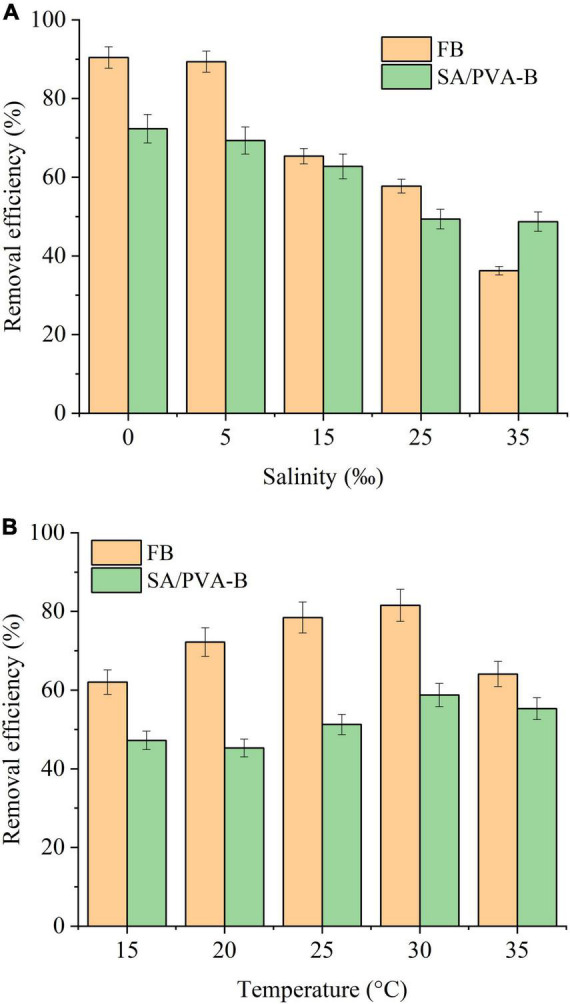
Effects of **(A)** salinity and **(B)** temperature on different immobilized biomaterials. FB, free *Ochrobactrum* sp. without any immobilization methods; SA/PVA-B, SA and PVA as base material to immobilize *Ochrobactrum* sp.

Microbial immobilization can resist the adverse environment. Salinity, as an important factor, affected the growth of microorganisms and osmotic pressure of cell membrane. In high-salinity environment, the growth of microorganisms was inhibited (Wang et al., 2017). The effect results of salinity on NH_4_^+^-N degradation are demonstrated in Figure 6A. For low salinity (lower than 5‰), free *Ochrobactrum* sp. group kept high NH_4_^+^-N removal efficiency (89.37–90.43%) and the degradation performance decreased to 36.24% when the salinity was up to 35‰. The phenomenon reflected that the nitrification process of *Ochrobactrum* sp. was inhabited under high salinity condition. However, the NH_4_^+^-N degradation efficiency was 69.32∼72.31% in 0 and 5‰ experiment groups, and the immobilization with BCCP produced a marked enhancement performance that displayed 12.47% higher than free *Ochrobactrum* sp. when the salinity increased to 35‰. Gao et al. (2020) found that immobilized materials owned a protective effect on bacteria in environments with high salinity and bacterial growth was inhibited when the salinity was higher than 15‰. Bacteria needed to obtain additional energy from the substrate to maintain cell activity in a high-salinity environment, and they could gradually adapt to high salinity environments over time (Moussa et al., 2005; Ge et al., 2019).

#### Effect of Temperature

Temperature is another key factor affecting microbial growth and enzyme activity, and the low temperature resistance for immobilized microorganisms is shown in Figure 6B. The NH_4_^+^-N removal efficiency of free or immobilized *Ochrobactrum* sp. groups increased with the temperature increasing from 15 to 30°C, while the efficiency decreased rapidly at 35°C. Compared with free bacteria group, microbial immobilization did not show its advantages, and its degradation efficiency was inferior to free *Ochrobactrum* sp. under different temperature conditions. For free bacteria group, 81% degradation efficiency was achieved at 30°C, which was 20% more than immobilized *Ochrobactrum* sp. group. For AOB, the optimal temperature is 30°C and bacteria grow perfectly at this temperature (Huang et al., 2017). When the temperature was lower than the optimal temperature, it affected the enzymatic reaction of cells and limited the growth rate of bacteria (Serra-Maia et al., 2016; Binnal and Babu, 2017; Huang et al., 2017; Manhaeghe et al., 2019). On the contrary, higher temperature could inactivate certain proteins in cell, reduced the activity of the microorganism, and even led to cell death (Ras et al., 2013; Serra-Maia et al., 2016; Nwoba et al., 2019). In this study, immobilized *Ochrobactrum* sp. did not play an effective role in resisting low temperature, but protected the *Ochrobactrum* sp. from the changes in salinity. The reason might be that as SA and PVA are the embedding materials of immobilized *Ochrobactrum* sp., the dense protective layer formed by them could buffer the salt concentration of microbial layer on the surface of BCCP, but could not block the impact of low temperature.

## Conclusion

The optimum calcined parameters of CBBP were calcined temperature 400°C, heating rate 20°C min^–1^, and holding time 2 h, and the composite particle owned better adsorption performance with 38.75% NH_4_^+^-N removal efficiency and 0.492 mg g^–1^ adsorption capacity. SA/PVA was more suitable as embedding material and jointed with BCCP (carrier) adsorbing NH_4_^+^-N, which was then degraded by *Ochrobactrum* sp. with the degradation efficiency of 79.39% at 168 h. Immobilizing *Ochrobactrum* sp. could protect the strain from high salt concentration to achieve the exceeding degradation efficiency than free bacteria; however, it could not block the impact of low temperature.

## Data Availability Statement

The original contributions presented in the study are included in the article/supplementary material, further inquiries can be directed to the corresponding author/s.

## Author Contributions

PS contributed to the data curation, methodology, and writing original draft, review, and editing. XH designed all the experiments, and revised and examined the manuscript. YX reviewed and edited the manuscript. WLD, JY, and JB contributed to the data curation and investigation. WYD interpreted the data and provided the resources. All authors read and approved the final manuscript.

## Conflict of Interest

The authors declare that the research was conducted in the absence of any commercial or financial relationships that could be construed as a potential conflict of interest.

## Publisher’s Note

All claims expressed in this article are solely those of the authors and do not necessarily represent those of their affiliated organizations, or those of the publisher, the editors and the reviewers. Any product that may be evaluated in this article, or claim that may be made by its manufacturer, is not guaranteed or endorsed by the publisher.
